# Androgen Receptor: Clinical Importance in Breast Cancer Patients Receiving CDK 4/6 Inhibitor Treatment

**DOI:** 10.3390/medicina61081464

**Published:** 2025-08-14

**Authors:** Seray Saray, Tufan Yılmaz, Hüseyin Kanmaz, Yakup İriağaç

**Affiliations:** 1 Department of Medical Oncology, Balikesir Ataturk City Hospital, Balikesir 10100, Turkey; 2 Department of Pathology, Balikesir Ataturk City Hospital, Balikesir 10100, Turkey

**Keywords:** androgen receptor, breast cancer, CDK 4/6 inhibitor

## Abstract

*Background and Objectives:* The effect of AR expression on prognosis in hormone receptor-positive her2-negative breast cancer is controversial. There are studies showing that AR is a treatment target, a mechanism of resistance to endocrine treatments, and a prognostic indicator in these patients whose standard treatment is a CDK 4/6 inhibitor added to endocrine treatment. We aimed to investigate the effect of AR, the AR/ER ratio, and the AR/PR ratio on CDK4/6 inhibitor treatment response in breast cancer, as well as their effects on PFS, and to validate the hypothesis that AR is a target for research. *Materials and Methods:* Patients who were diagnosed with metastatic hormone receptor-positive her2-negative breast cancer and received cdk4/6 inhibitor + aromatase inhibitor in first-line therapy were included in this study conducted at Balıkesir Atatürk City Hospital. The tru-cut biopsy samples of the patients were evaluated immunohistochemically for AR, ER, and PR. Kaplan–Meier analysis was used to calculate the estimated median survival in PFS analyses, and the variables were compared with the Log-Rank test. Receiver Operating Characteristic (ROC) analysis was applied to determine the ideal cut-off. Cox regression analysis was used in univariate survival models, and the multivariate model was established with the “Forward: Likelihood Ratio (LR)” method. Hazard ratios (HRs) were also calculated as 95% confidence intervals (95% CIs). A *p* value below 0.05 was accepted for statistical significance. *Results:* In total, 41 patients were included in the study, and 73% (n = 30) of the patients were AR-positive. Increased AR (HR 1.014; 95% CI: 1.002–1.026; *p* = 0.023) was an unfavorable prognostic indicator. In our study, being ≥55 years old, being postmenopausal, not having visceral metastasis, having a non-IDC histology, having a low AR level (<50%), having an AR/ER ratio < 0.74, and having an AR/PR ratio < 1.00 were found to be associated with longer PFS. All factors were evaluated with univariate Cox regression analysis. Increasing AR (HR 1.014; 95% CI: 1.002–1.026; *p* = 0.023) was an unfavorable prognostic marker. Having an AR/ER ratio ≥ 0.74 (HR: 2.522; 95% CI: 1.004–6.336; *p* = 0.049) and having AR/PR ≥ 1 (HR: 2.659; 95% CI: 1.029–6.869; *p* = 0.043) were negative prognostic indicators. *Conclusions:* Our results were consistent with the literature and demonstrated the value of the androgen receptor as a therapeutic target, a mechanism explaining resistance to endocrine therapy, and an adverse prognostic indicator for creating resistance to endocrine therapy in breast cancer.

## 1. Introduction

Despite advances in early diagnosis and treatment that have led to decreasing mortality rates, breast cancer remains the most frequently diagnosed cancer in women worldwide and the second leading cause of cancer-related deaths [1,2].

Among the subtypes of breast cancer, the hormone receptor (HR)-positive/human epidermal growth factor receptor 2 (HER2)-negative luminal subtype is the most common, accounting for approximately 70% of all cases [3,4]. Luminal A breast cancer is defined as estrogen receptor (ER)- and/or progesterone receptor (PR)-positive, HER2-negative, and having low Ki-67 expression, indicating a lower proliferative rate [5]. In contrast, luminal B is also ER- and/or PR-positive and HER2-negative, but characterized by high Ki-67 expression, which is associated with higher proliferation and a worse prognosis [5].

Cyclin-dependent kinase 4/6 (CDK4/6) is an important regulator of the cell cycle that acts by forming a complex with cyclin D [6]. The CDK 4/6-cyclin D pathway triggers cell cycle progression in response to estrogen signaling [7]. The cyclin D-CDK 4/6-inhibitor of the CDK4 (INK4)–retinoblastoma (Rb) pathway controls cell cycle progression by regulating the G1-S checkpoint [8]. CDK 4/6 inhibition in combination with endocrine therapy is the standard treatment used worldwide in luminal A and B subtype breast cancer [9]. The cyclin-dependent kinase 4 and 6 inhibitors palbociclib, ribociclib, and abemaciclib have been studied in combination with endocrine therapy (ET) in metastatic breast cancer (mBC) and have been shown to prolong progression-free survival (PFS); ribociclib also provides an overall survival (OS) advantage compared to endocrine monotherapy [10]. However, resistance to these treatments may develop through mechanisms such as RB loss with mutation in the retinoblastoma 1 (RB1) gene, E2F amplification, PI3K/AKT/mTOR signaling pathway mutations, and androgen receptor (AR) overexpression [3].

AR is a steroid hormone receptor that acts as a ligand-dependent transcription factor [11]. The androgen receptor (AR) is expressed in over 70% of breast cancers, suggesting its potential as a novel biomarker and therapeutic target for breast cancer patients [12]. In HR-positive breast cancer, expression is associated with both a favorable prognosis and resistance to endocrine therapy [13,14]. It is also considered a potential additional marker for predicting response to endocrine treatment [13,14]. However, due to conflicting findings across studies, the clinical value of AR as a biomarker and therapeutic target in breast cancer remains uncertain [12]. In the literature, the AR/ER ratio has been investigated to evaluate the role of AR, and it has been identified as an independent predictor of overall survival (OS) and disease-free survival (DFS) in breast cancer patients [15]. However, some studies have reported that the AR/ER ratio does not reliably predict the benefit derived from endocrine therapy [16].

In this study, we aimed to investigate the effects of androgen receptor (AR) expression, as well as the AR/ER and AR/PR ratios, on responses to CDK4/6 inhibitor treatment and progression-free survival (PFS) in breast cancer patients. Additionally, we sought to explore the relationships between these markers and the clinicopathological characteristics of the patients. Furthermore, this study aimed to validate the hypotheses of AR as a potential therapeutic target and a prognostic marker in breast cancer.

## 2. Materials and Methods

### 2.1. Patients

This study included patients who were diagnosed at the Balıkesir Atatürk City Hospital of Health Sciences University between 1 March 2019 and 1 January 2024, and who were stage 4 according to the TNM Classification of Malignant Tumors at the time of diagnosis. The patients’ AR, PR, ER, ki-67, and nuclear grade parameters were obtained with tru-cut biopsy samples taken at the time of diagnosis. Other data were obtained retrospectively from patient records.

The study included patients who were Her2-negative, 18 years of age or older, and had sufficient tumoral tissue for tru-cut biopsy. Patients who were Her2-positive, those who received chemotherapy outside of standard treatment, and those for whom sufficient tissue samples could not be obtained for staining were not included in the study.

PFS was defined as the time from the diagnosis of metastatic breast cancer to disease progression in this group of patients who received first-line treatment at the metastatic stage.

### 2.2. Pathological Evaluation

Paraffin-embedded tissue samples, previously prepared with tru-cut biopsy and Hematoxylin–Eosin-stained preparations, were obtained from the hospital’s pathology tissue archive. Sections were prepared by two pathology technicians using a microtome. Immunohistochemical (IHC) staining was performed, and the sections were placed on the BenchMark XT device.

The following antibodies were used in the staining procedures:

- ER: SP1 (Ventana).
- PR: 1E2 (Ventana).
- AR: SP107 (Ventana).
- HER-2: Anti-HER-2/neu; 4B5 (Ventana).
- Ki-67: 30-9 (Ventana).

Normal breast tissue was used as an external control. The IHC staining results were evaluated with an Olympus BX51 microscope by a pathologist experienced in breast cancer, and percentage counts were calculated. The evaluation was performed according to the College of American Pathologists (CAP) Breast Biomarker Template 2020 (Template for Reporting Results of Biomarker Testing of Specimens From Patients With Carcinoma of the Breast) guidelines [17].

The HER-2 staining score was determined in accordance with ASCO/CAP guidelines [18]. Accordingly,

- 0 or 1+: HER-2 was considered negative.
- 2+: HER-2 was classified as negative or positive by the fluorescence in situ hybridization (FISH) test.
- 3+: HER-2 was evaluated as positive.

Luminal-type breast cancer was defined as HER-2 negative patients with an ER or PR expression of 1% and above [19]. Samples with 1% to 100% of tumor nuclei positive for ER or progesterone receptor (PgR) were interpreted as positive [18]. As regards AR expression, tissues showing ≥1% positive tumor cells in their nucleus were defined as positive [20].

### 2.3. Statistical Analyses

SPSS Statistics software 24 (SPSS Inc., Chicago, IL, USA, III) was used in all the statistical analyses. Kaplan–Meier analysis was used to calculate the estimated median survival in PFS analyses, and the variables were compared with the Log-Rank test. The distributions of the data were analyzed with Kolmogorov–Smirnov; according to these results, correlation analyses were performed with Spearman correlation analysis, and the *p* value and correlation coefficient (R) were determined. Receiver Operating Characteristic (ROC) analysis was applied to determine the ideal cut-off. For variables that did not reach statistical significance, the median value, a method commonly accepted in the literature, was used as a reference point. Based on the median, these variables were categorized into two groups: high and low [21,22].

Cox regression analysis was used in univariate survival models, and the multivariate model was established using the “Forward: Likelihood Ratio (LR)” method. Variables that were statistically significant in the univariable Cox regression analysis (*p* < 0.05) were included in the multivariable model using a forward stepwise selection method, and the final model was presented accordingly. AR/ER and AR/PR were calculated separately. A value of 0.01 was accepted as a mathematical error for 0 values. The hazard ratio was also calculated as a 95% confidence interval (95% CI). A *p* value below 0.05 was accepted for statistical significance.

## 3. Results

In total, 41 patients were included in the study. The median age of the patients was 55 (min: 32–84). The median ER level of the patients was 90% (min: 20%; max: 95%), and their median PR level was 60% (min: 0%; max: 90%). Median AR was determined as 30% (min: 5%; max: 80%), and median ki-67 was also determined as 30% (min: 5%; max: 80%). In total, 73% (n = 30) of patients were AR-positive, 70.7% (n = 29) were postmenopausal, and 80.5% (n = 33) had no history of chemotherapy. Before treatment, 36.6% (n = 15) of patients had visceral metastases and 80.5% (n = 33) had bone metastases. In total, 51.2% (n = 21) of patients were treated with ribociclib and 48.8% (n = 20) with palbociclib (Table 1).

**Figure 1:**
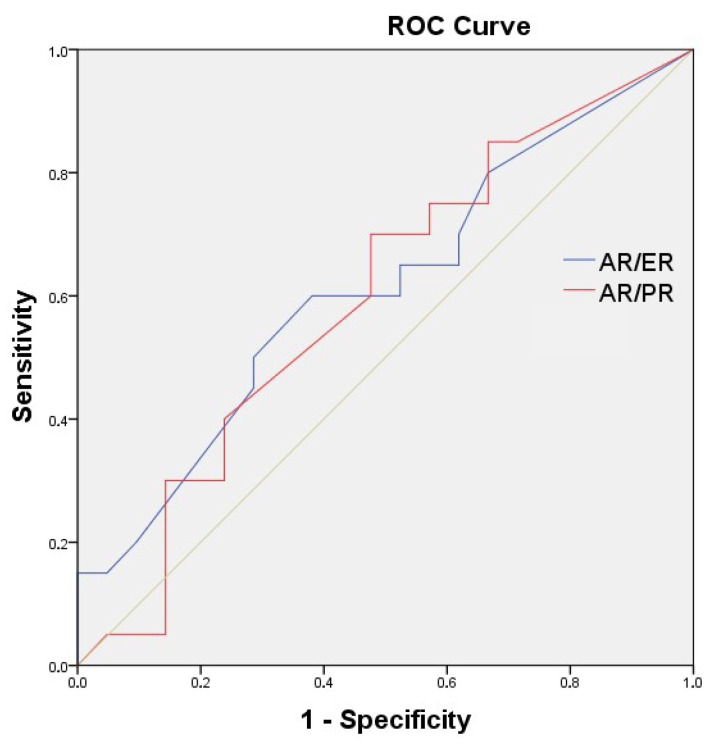
ROC curve analysis for AR/ER and AR/PR.

**Figure 2:**
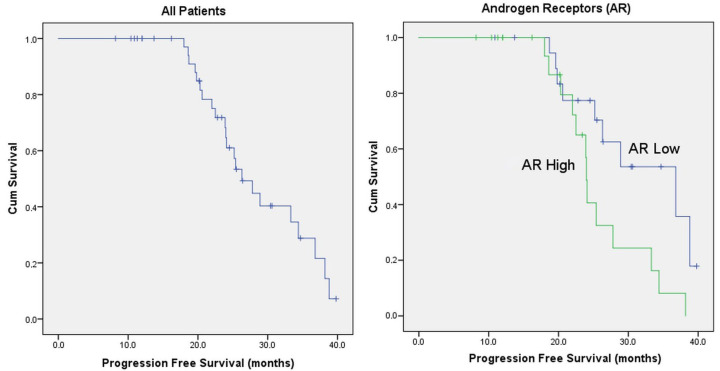

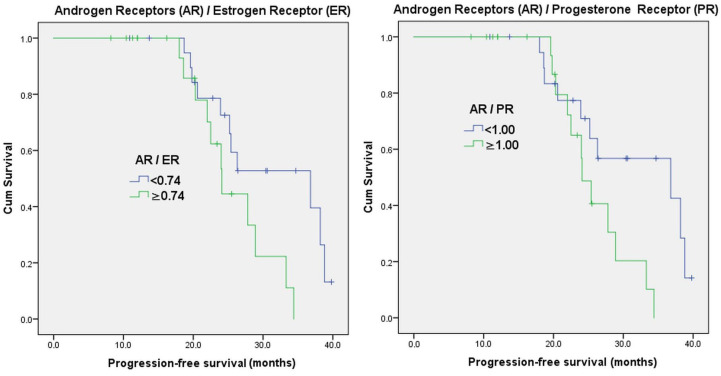
Survival graphs of the general population and AR, AR/ER, and AR/PR variables.

The ideal cut-off could not be determined by ROC curve analysis for AR/ER and AR/PR (AUC for AR/ER = 0.613; 95% CI: 0.439–0.787; *p* = 0.215; and AUC for AR/PR = 0.596; 95% CI: 0.420–0.773; *p* = 0.291) (Figure 1). Therefore, median values were taken for survival (AR/ER median = 0.74; AR/PR median = 1.00). The estimated median PFS for all patients was 26.3 months. AR was divided into 2 according to the median value (<50%/≥50%). The estimated median PFS time for those with a low AR was 36.8 months, whereas that for those with a high AR was 24.0 months (*p* = 0.025). The estimated median PFS for AR/ER ≥ 0.74 was 24.1 months, while it was 36.8 months for AR/ER < 0.74 (*p* = 0.042). The estimated median PFS for AR/PR < 1.00 was 38.8 months, and 24.1 months for AR/PR ≥ 1.00. (*p* = 0.036). The median PFS was 33.3 months in postmenopausal patients and 22.0 months in pre-perimenopausal patients (*p* = 0.039) (Table 1 and Figure 2).

When age was examined with correlation between AR, ER, and PR, there was a positive correlation only between AR and PR (r = 0.395; *p* = 0.011). There was no correlation between the others (Table 2).

All factors were evaluated with univariate Cox regression analysis. Increasing age (HR: 0.955; 95% CI: 0.914–0.999; *p* = 0.044) was a favorable prognostic marker. Increasing AR (HR: 1.014; 95% CI: 1.002–1.026; *p* = 0.023) was an unfavorable prognostic marker. Having visceral metastasis (HR: 3.769; 95% CI: 1.160–12.248; *p* = 0.027), having an AR/ER ratio ≥ 0.74 (HR: 2.522; 95% CI: 1.004–6.336; *p* = 0.049), and having AR/PR ≥ 1 (HR: 2.659; 95% CI: 1.029–6.869; *p* = 0.043) were negative prognostic indicators (Table 3).

All factors that were significant with univariate analysis provided a predictive model feature for AR increase (HR: 1.015; 95% CI: 1.003–1.027; *p* = 0.018) and visceral metastasis (HR: 4.065; 95% CI: 1.245–13.280; *p* = 0.020) when evaluated with multivariate analysis. In an analysis where AR was not included, the AR/PR ratio (HR: 2.852; 95% CI: 1.104–7.368; *p* = 0.030) and visceral metastasis (HR: 4.204; 95% CI: 1.283–13.771; *p* = 0.018) provided a predictive model feature (Table 3).

## 4. Discussion

In this study, the expression rate of androgen receptor (AR) in the hormone receptor-positive/HER2-negative breast cancer patient population was 73%, which is consistent with values reported in the literature [12]. Anestis et al. stated in their article that, due to the high expression of androgen receptor (AR) in breast cancer, AR could serve as a therapeutic target [12]. They also highlighted that preliminary clinical studies using AR-targeted drugs originally approved for prostate cancer have shown promising results in AR-positive breast cancer patients [12]. Notably, Hickey et al. demonstrated in their study that combining AR agonists with standard endocrine therapy and CDK4/6 inhibitors in ER-positive breast cancer enhanced therapeutic responses and decreased treatment resistance [23]. They attributed these effects to AR activation, the suppression of ER-regulated cell cycle genes, and the upregulation of AR target genes, including tumor suppressors [23].

In our study, being ≥55 years old, being postmenopausal, not having visceral metastasis, a non-IDC histology, having a low AR level (<50%), an AR/ER ratio < 0.74, and an AR/PR ratio < 1.00 were found to be associated with longer PFS. In the study of Elebro et al., AR negativity predicted early treatment failure with AI but not with tamoxifen [24]. In the study of Cochrane et al., in a cohort of 192 ER + breast cancer women, it was seen that a high AR/ER ratio (≥2.0) increased the risk of failure more than fourfold while using tamoxifen; the researchers stated that a high AR/ER ratio may negatively affect the response to endocrine therapy in breast cancer, and that AR may activate protumorigenic pathways in breast cancers in the absence of estrogen in a postmenopausal patient receiving AI treatment [25]. De Amicis et al. found increased AR and decreased ER-α mRNA in tamoxifen-resistant tumors with gene expression profiling [26]. They defined AR overexpression as a new mechanism of hormone resistance and thus stated that AR could be a new clinical therapeutic target in human breast cancers [26]. Rangel et al. reported in their study that an AR/ER ratio ≥ 2 defines a subgroup of patients with aggressive biological features, that this is a marker for poor prognosis, and that a significant number of cases with AR/ER ≥ 2 may be non-luminal tumors [27].

In our study, the significantly shorter progression-free survival (PFS) observed in the group with high AR levels and an AR/ER ratio ≥ 0.74, compared to the group with a ratio below 0.74, aligns with the previous literature indicating a poor prognosis. Anestis et al. explained this phenomenon by suggesting that AR competes with ER in ER-positive breast cancer, leading to impaired ER transcription and increased apoptosis [12]. The interaction between AR, ER, and their ligands is complex; the possible conversion of androgens to estrogens may further influence cell proliferation either positively or negatively [28]. While AR is generally associated with a favorable prognosis in hormone receptor-positive breast cancer, as reported by Khan et al., it also contributes to resistance to endocrine therapy [13]. Rechoum et al. demonstrated that AR binding to estrogen receptor response elements (EREs) can reduce estrogen’s proliferative effects, whereas ER binding to androgen response elements (AREs) may have the opposite impact [29]. This mechanism may explain the potential role of AR in resistance to standard hormonal therapies [29].

Work by Young-Chae et al. in human prostate epithelial cell lines suggested that AR-mediated growth suppression involves cyclin D1 mRNA degradation, the transcriptional repression of cyclin D2 and CDK4/6, and the transcriptional activation of CDKN1A, all of which contribute to reduced CDK4/6 activity [30]. Similar mechanisms may explain the high AR level in our patients receiving standard hormonal therapy + CDK 4/6 inhibitor treatment and the low PFS observed in the group with an AR/ER ratio ≥ 0.74 with treatment resistance. The unclear relationship between AR positivity and prognosis may be explained by findings from Kolyvas et al., who reported that AR positivity determined by AR gene expression does not accurately reflect AR activity [11].

The limitations of our study include the lack of molecular profiling and the fact that it was conducted in a single center with a relatively small number of patients. However, a post hoc power analysis for the AR/ER ratio (≥0.74 vs. <0.74), based on a hazard ratio of 2.52 and a sample size of 41 (21 vs. 20 patients), yielded an estimated statistical power of approximately 80% (α = 0.05), indicating sufficient power to detect the observed effect. Due to the short follow-up period, PFS analyses showing the first-line treatment response time instead of overall survival are also among the limitations. Nevertheless, our study yielded results consistent with the literature, indicating that androgen receptor can be used as a therapeutic target in breast cancer, a mechanism explaining resistance to endocrine therapy, and a prognostic marker. Since molecular tests cannot be applied to every patient, especially in developing countries, the clinical importance of AR expression, which is cheaply and easily measured with IHC, will gain more meaning.

## 5. Conclusions

In our study, the AR expression rate was 73% among the HR-positive/HER2-negative breast cancer patient population. Furthermore, a low AR level (<50%), an AR/ER ratio below 0.74, and an AR/PR ratio below 1.00 were associated with longer progression-free survival (PFS). These findings, consistent with the literature, highlight the androgen receptor’s significance as a therapeutic target, a potential mechanism underlying resistance to endocrine therapy, and a prognostic marker in breast cancer.

## Figures and Tables

**Table 1:** Clinical and pathological characteristics of patients and median progression-free survival (PFS).

		n	(%)	Median PFS (Month)	*p* *
Age	<55	19	46.3%	25.4	0.036
	≥55	22	53.7%	34.4	
AR	Low (<%50)	20	48.8%	36.8	0.025
	High (≥%50)	21	51.2%	24.0	
AR/ER ratio	<0.74	21	51.2%	36.8	0.042
	≥0.74	20	48.8%	24.1	
AR/PR ratio	<1.00	20	48.8%	36.8	0.036
	≥1.00	21	51.2%	24.1	
Menopause status	Pre-peri	12	29.3%	22.0	0.039
	Post	29	70.7%	33.3	
Breast cancer surgery	Yes	20	48.8%	26.3	0.641
	No	21	51.2%	24.1	
Chemotherapy	No	33	80.5%	25.2	0.059
	Yes	8	19.5%	28.9	
Diagnostic stage	Stage (1-2-3)	11	26.8%	24.1	0.245
	Stage 4	30	73.2%	27.8	
Adjuvant hormonotherapy	No	30	73.2%	27.8	0.245
	Yes	11	26.8%	24.1	
Lymph node metastasis	No	13	31.7%	28.9	0.701
	Yes	28	68.3%	25.4	
Visceral metastasis	No	26	63.4%	28.9	0.018
	Yes	15	36.6%	20.6	
Bone metastasis	No	8	19.5%	34.4	0.682
	Yes	33	80.5%	25.4	
Radiotherapy	No	20	48.8%	34.4	0.094
	Yes	21	51.2%	23.9	
Grade	1-2	26	63.4%	27.8	0.267
	3	15	36.6%	22.0	
Histology	Ductal	34	82.9%	25.2	0.048
	Others	7	17.1%	38.2	
Treatment	Ribociclib	21	51.2%	24.1	0.091
	Palbociclib	20	48.8%	34.4	

*p* * for PFS. Kaplan–Meier analysis. Obtained by Log-Rank comparison test. Abbreviations: AR: Androgen receptor; AR/ER: Androgen receptor/estrogen receptor; AR/PR: Androgen receptor/progesterone receptor.

**Table 2:** Correlation analysis of AR, ER, age, and Ki-67 with each other.

	AR		ER		PR		Ki-67	
	R	*p*	R	*p*	R	*p*	R	*p*
Age	−0.093	0.564	0.102	0.528	−0.028	0.861	−0.136	0.398
AR			−0.116	0.472	0.395	0.011	−0.108	0.500
ER					0.189	0.235	0.138	0.388
PR							−0.067	0.678

Abbreviations: AR: Androgen receptor; ER: Estrogen receptor; PR: Progesterone receptor.

**Table 3:** Univariate analysis and multivariate modeling of factors affecting survival of patients using CDK 4-6.

Variable	Category	HR (95% CI)	*p*
Age	Continuous	0.955 (0.914–0.999)	0.044
ER	Continuous	1.002 (0.979–1.025)	0.864
PR	Continuous	1.003 (0.990–1.015)	0.690
AR	Continuous	1.014 (1.002–1.026)	0.023
AR/ER ratio	0.74</≥0.74	2.522 (1.004–6.336)	0.049
AR/PR ratio	1.00</≥1.00	2.659 (1.029–6.869)	0.043
Ki-67	Continuous	1.008 (0.986–1.030)	0.485
Grade	1-2/3	1.712 (0.656–4.470)	0.272
Menopausal status	Pre-peri/post	0.739 (0.550–0.995)	0.046
Breast cancer surgery	No/yes	1.223 (0.525–2.849)	0.642
Chemotherapy	No/yes	0.796 (0.321–1.971)	0.621
Diagnostic stage	1/2/3/4	0.822 (0.566–1.194)	0.303
Adjuvant hormonotherapy	No/yes	1.709 (0.685–4.263)	0.251
Lymph node metastasis	No/yes	1.195 (0.482–2.964)	0.701
Visceral metastasis	No/yes	3.769 (1.160–12.248)	0.027
Bone metastasis	No/yes	1.259 (0.418–3.789)	0.682
Radiotherapy	No/yes	2.088 (0.868–5.023)	0.100
Histology	Ductal/Others	0.303 (0.087–1.053)	0.060
Treatment	Ribo/palbo	0.460 (0.183–1.157)	0.099
Multivariate Model 1 *			
AR	Continuous	1.015 (1.003–1.027)	0.018
Visceral metastasis	No/yes	4.065 (1.245–13.280)	0.020
Multivariate Model 2 **			
AR/PR ratio	1.00</≥1.00	2.852 (1.104–7.368)	0.030
Visceral metastasis	No/yes	4.204 (1.283–13.771)	0.018

Abbreviations: AR: Androgen receptor; ER: Estrogen receptor; PR: Progesterone receptor; HR: Hazard ratio. * Variables that were found to be statistically significant in the univariate analysis (Age, AR, AR/ER ratio, AR/PR ratio, menopausal status, and visceral metastasis) were included in the multivariate model using the “Forward: Likelihood Ratio (LR)” stepwise method. The final step of this analysis is presented as Model 1. ** When AR was excluded from the analysis, the remaining variables (Age, AR/ER ratio, AR/PR ratio, menopausal status, and visceral metastasis) were re-analyzed using the same method, and the final step is presented as Model 2.

## Data Availability

Although not publicly available, the datasets created and/or analyzed during the current study are available from the corresponding author upon justifiable request.

## Abbreviations

The following abbreviations are used in this manuscript:

CDK	Cyclin-Dependent Kinase
HR	Hormone Receptor
HER2	Human Epidermal Growth Factor Receptor 2
ER	Estrogen Receptor
PR	Progesterone Receptor
ET	Endocrine Therapy
MBC	Metastatic Breast Cancer
PFS	Progression-Free Survival
OS	Overall Survival
RB1	Retinoblastoma
AR	Androgen Receptor
IHC	Immunohistochemistry
CAP	College Of American Pathologists
ROC	Receiver Operating Characteristic
LR	Likelihood Ratio
